# Nutrition-modulated, subtype-specific risk factors for catheter-related bloodstream infections in hospitalized patients with intestinal failure

**DOI:** 10.3389/fnut.2025.1705357

**Published:** 2025-11-20

**Authors:** Minyi Zhu, Sirui Liu, Yi Wu, Qin Pan, Yupeng Zhang, Gulisudumu Maitiabula, Xuejin Gao, Xinying Wang

**Affiliations:** 1 Clinical Nutrition Service Center, Department of General Surgery, Nanjing Jinling Hospital, Affiliated Hospital of Medical School, Nanjing University, Nanjing, China; 2 Department of General Surgery, Jinling Clinical Medical College, Nanjing University of Chinese Medicine, Nanjing, China; 3 Department of General Surgery, Jinling Hospital, Southeast University School of Medicine, Nanjing, China

**Keywords:** parenteral nutrition, intestinal failure, catheter-related bloodstream infections, nutritional support, enteral nutrition

## Abstract

**Background and aims:**

Parenteral nutrition is essential for patients with intestinal failure but predisposes them to catheter-related bloodstream infection, a serious complication threatening survival. Prior research emphasizes catheter management, while the role of parenteral nutrition delivery strategies—particularly energy proportion and nutrient composition—remains poorly understood. This study investigates catheter-related bloodstream infection risk factors, focusing on parenteral nutrition energy supply and formulation, and evaluates subtype-specific susceptibilities in hospitalized patients with intestinal failure.

**Methods:**

This retrospective study analyzed 321 hospitalized patients with intestinal failure, encompassing 9,365 catheter-days. catheter-related bloodstream infection incidence was calculated per 1,000 catheter-days. Univariate and multivariate logistic regression and Cox proportional hazards regression identified independent risk factors. Stratified analyses identified subtype-specific risks, and hospital stay length and health economic outcomes were assessed.

**Results:**

The overall catheter-related bloodstream infection incidence was 7.048 per 1,000 catheter-days, significantly exceeding benchmarks. Key independent risk factors were parenteral nutrition calories > 60% of resting energy expenditure (OR = 3.808, HR = 2.055), lymphocytopenia (< 1 × 10^9^/L; OR = 6.047), high calorie-to-nitrogen ratio (≥ 100 kcal/g N; OR = 2.118), neutropenia (< 1.5 × 10^9^/L; HR = 2.573), and hypertension (OR = 4.981). Subtype-specific modulation was evident. Catheter-related bloodstream infection significantly prolonged hospitalization by nearly 2 weeks and increased inpatient costs.

**Conclusion:**

Optimizing parenteral nutrition strategies, particularly by minimizing duration of high proportion of energy supply by parenteral nutrition through progressive enteral nutrition, is critical to reduce catheter-related bloodstream infection. Administering a low calorie-to-nitrogen ratio parenteral nutrition formula with immunonutrients is essential in unstable type I/II patients, while type III requires emphasis on blood pressure management. Universal multidrug-resistant pathogen vigilance is needed.

## 1 Introduction

Intestinal failure (IF) is clinically defined as the reduction of gut function below the minimum level necessary for adequate absorption of macronutrients and/or water-electrolytes, necessitating intravenous supplementation to maintain health and/or growth. Based on standardized criteria encompassing disease onset, metabolic status, and clinical prognosis, intestinal failure is classified into three distinct categories: Type I: Characterized by acute, short-term, and typically self-limiting conditions; Type II: Representing prolonged acute conditions, predominantly occurring in metabolically unstable patients who require comprehensive multidisciplinary care and weeks to months of intravenous support; Type III: Denoting chronic conditions in metabolically stable patients necessitating intravenous supplementation for extended periods ranging from months to years (1).

The dependence on parenteral nutrition (PN) in IF patients renders catheter-related bloodstream infections (CRBSI) one of the most life-threatening PN-associated complications (2). Clinical management of CRBSI typically necessitates immediate removal of virtually all intravenous catheters to prevent progression to sepsis. However, repeated catheter replacements may ultimately lead to loss of venous access, thereby compromising this vital source of life-sustaining nutrition. Early identification of risk factors for CRBSI development in IF patients is crucial for reducing infection rates and improving patients’ quality of life.

Previous studies on risk factors for CRBSI in patients with type III intestinal failure receiving HPN have primarily focused on catheter management, intestinal anatomy, and antibiotic lock therapy (3–6). Several critical knowledge gaps persist in clinical practice: (1) whether the composition of PN formulas and delivery strategies per se have a direct impact on the risk of CRBSI; (2) whether different IF subtypes exhibit distinct infectious mechanisms and thus require tailored nutritional therapeutic approaches; and (3) whether the risk factors for CRBSI in hospitalized IF patients warrant different considerations compared to those in HPN settings.

PN formulation must be tailored to the specific needs, metabolic status, and contraindications of IF patients. Immunonutrients such as fish oil and glutamine support immune barrier function and cellular metabolism (7), while other nutritional factors including caloric distribution, nitrogen availability, and glucose-lipid ratio may directly affect infection resistance. Furthermore, hospitalized IF patients likely face substantially higher CRBSI rates compared to HPN settings due to multiple risk factors including high nosocomial pathogen exposure, frequent catheter manipulation, and concurrent invasive procedures.

To address these research gaps, this study investigate CRBSI risk factors in hospitalized patients with all types of IF. Furthermore, we analyzed the subtype-specific susceptibility to various risk factors and conducted a health economic evaluation, highlighting the essential role of tailored nutrition support in modulating clinical outcomes.

## 2 Material and methods

### 2.1 Study population

**FIGURE 1:**
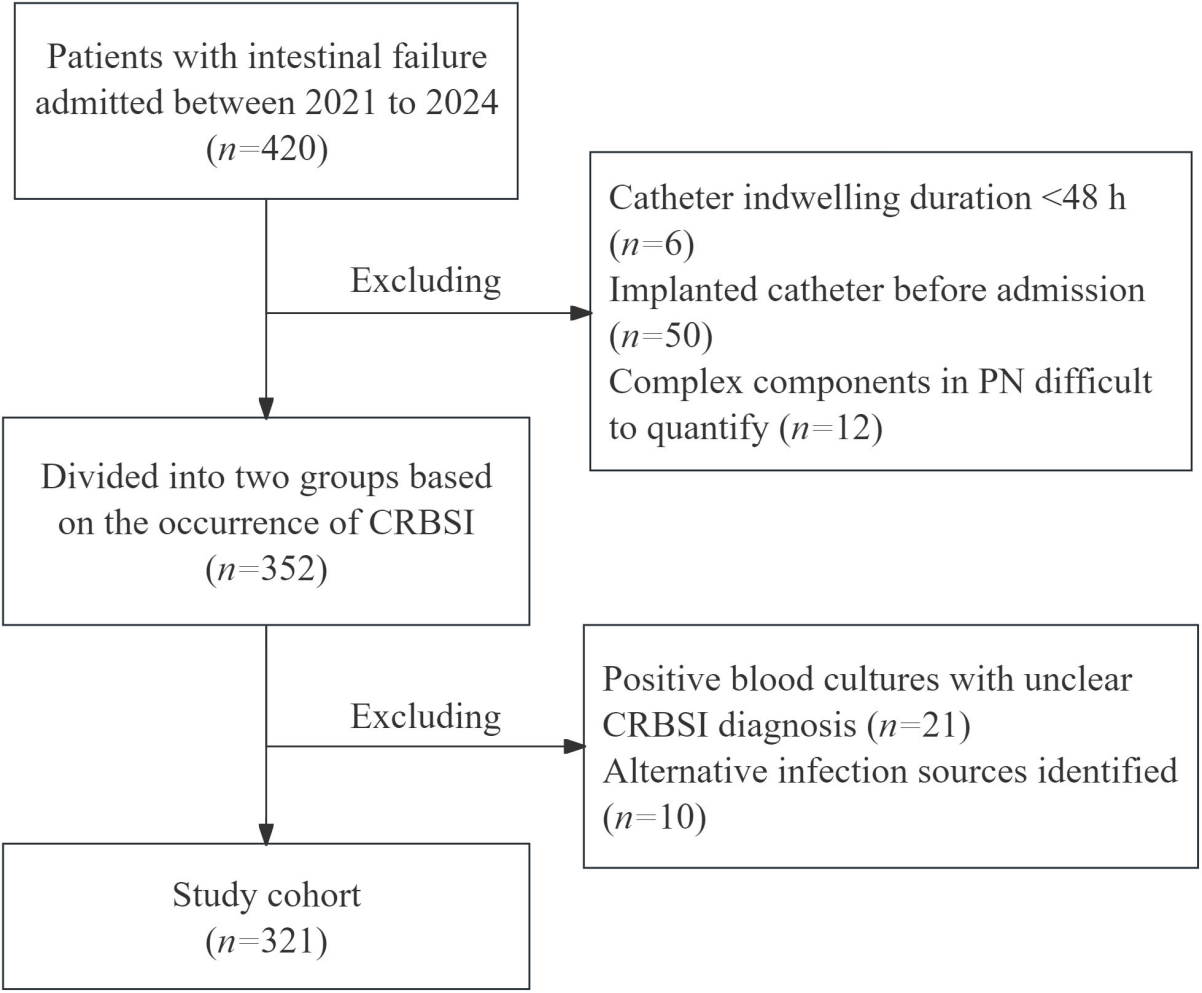
Flow-chart of the study.

This single-center retrospective study enrolled 321 IF patients admitted between 2021 and 2024 to analyze the risk factors for CRBSI. All included patients met the ESPEN 2023 diagnostic criteria for intestinal failure. Patients were excluded if they met any of the following criteria: Catheter indwelling duration < 48 hours; Implanted catheter before admission; Complex PN components difficult to quantify; Positive blood cultures with unclear CRBSI diagnosis; Alternative infection sources identified (Figure 1). Following hemodynamic stabilization, venous access devices were placed for PN administration, with prospective monitoring for CRBSI throughout hospitalization. The insertion and maintenance of all venous catheters adhered strictly to the Clinical Practice Guideline for the Prevention and Treatment of Central Line-Associated Bloodstream Infections by the National Health Commission of the People’s Republic of China. Our protocol comprised several key components: maximal sterile barrier precautions (utilizing sterile gowns, gloves, caps, masks, and large drapes) during catheter placement, skin antisepsis with 2% chlorhexidine gluconate, and a daily review of line necessity by the clinical team. Furthermore, post-insertion care, including all dressing changes, was conducted by certified nursing staff according to a standardized protocol integral to our intestinal failure patient care bundle.

According to the ESPEN guidelines, blood glucose should be maintained between 7 and 10 mmol/L during parenteral nutrition administration. For diabetic patients, a common recommendation is 1 unit of regular insulin per 10 grams of glucose. For non-diabetic patients who develop hyperglycemia, the suggested ratio is 1 unit of regular insulin per 20 grams of glucose (8).

### 2.2 Definition of CRBSI

The definition of catheter-related bloodstream infection (CRBSI) in this study adhered to the clinical practice guidelines issued by the Infectious Diseases Society of America (IDSA). Patients were diagnosed with CRBSI if they fulfilled both of the following criteria during the study period: (1) clinical manifestations of sepsis or bacteremia unexplained by alternative causes, including fever (>38.3°C), hypotension, or rigors; and (2) concordant microbial growth of at least one identical pathogen from paired cultures of the catheter tip and peripheral venous blood (1).

### 2.3 Assessment of nutritional variables and data collection

At admission, baseline data were collected through clinical assessment, anthropometric measurements and nutritional evaluation. All patients underwent nutritional risk screening using the Nutritional Risk Screening 2002 (NRS-2002) tool. Diagnosis and severity assessment of malnutrition were performed according to the Global Leader-ship Initiative on Malnutrition (GLIM) criteria. The most recent serum biomarker data obtained within 2 days before catheter insertion were included in the analysis. The following factors were further analyzed: CRBSI pathogens, PN administration frequency, the proportion of PN-derived energy, PN nutrient composition (non-protein calorie-to-nitrogen ratio, glucose-to-lipid ratio, and supplementation with Omega-3 polyunsaturated fatty acids or glutamine), type of venous catheter, length of hospital stay, and hospitalization costs. Resting energy expenditure (REE) was measured via indirect calorimetry on the first day of admission and reassessed weekly.

The PN energy proportion relative to REE (PN/REE) was calculated as:


$$\mathrm{PN}/\mathrm{REE}=\frac{\begin{array}{c} \mathrm{Daily}\mathrm{total}\mathrm{energy}\mathrm{from}\mathrm{PN}\left(\mathrm{kcal}\right)\times \\ \mathrm{Weekly}\,\mathrm{administration}\,\mathrm{frequency} \end{array}}{7\times \mathrm{Weekly}\,\mathrm{REE}\,\left(\mathrm{kcal}\right)}$$

The daily PN volume per week was determined as:


$$\mathrm{PN}\,\mathrm{volume}\,\left(\text{ml}/\text{d}\right)=\frac{\begin{array}{c} \mathrm{PN}\mathrm{volume}\mathrm{per}\mathrm{administration}\left(\text{ml}\right)\times \\ \text{Week}\,\mathrm{ly}\,\mathrm{PN}\,\mathrm{frequency} \end{array}}{7}$$

The non-protein calorie-to-nitrogen (C/N) ratio was defined as:


$$C/N\,\mathrm{ratio}\,\left(\mathrm{kcal}/g\right)=\frac{\mathrm{Non}-\mathrm{protein}\,\mathrm{calorie}\,\left(\mathrm{kcal}\right)}{\mathrm{Nitrogen}\,\mathrm{intake}\,\left(g\right)}$$

The glucose-to-lipid ratio was calculated as:


$$\mathrm{Glucose}-\mathrm{to}-\mathrm{lipid}\,\mathrm{ratio}=\frac{\mathrm{Calories}\,\mathrm{from}\,\mathrm{glucose}\,\left(\mathrm{kcal}\right)}{\mathrm{Calories}\,\mathrm{from}\,\mathrm{lipid}\,\left(\mathrm{kcal}\right)}$$

The PN/REE, weekly PN administration frequency, daily PN volume, glucose-to-lipid ratio, and non-protein calorie-to-nitrogen ratio were analyzed using the mean values during the venous catheter indwelling period.

While the direct link between PN volume and CRBSI risk is not well-established, high volumes prolong infusion time and serve as a surrogate for increased nutrient loads, potentially compromising immunity. We therefore hypothesized that high PN volume increases CRBSI risk. Patients were dichotomized by the cohort’s median weekly PN volume (>1650 vs. ≤ 1650 mL/day).

Furthermore, based on prior evidence that 40% enteral nutrition reverses PN-induced gut immune injury (9), we hypothesized that a higher ratio of PN to REE (>0.6) would predispose to immune dysfunction and CRBSI. Patients were accordingly stratified by a PN/REE ratio cutoff of 0.6.

The calorie-to-nitrogen ratio is a key nutritional parameter reflecting the balance between non-protein energy and nitrogen supply from amino acids. An appropriate ratio ensures that amino acids are utilized for protein synthesis rather than being catabolized for energy. An elevated ratio may indicate either insufficient amino acid availability for anabolic requirements or excessive non-protein calories, potentially compromising metabolic efficacy.

### 2.4 Statistical analysis

Statistical analyses were performed using R Studio software package (Version 4.2.1) and SPSS 26.0, along with Zstats v1.0.^1^ Continuous data were summarized as mean ± standard deviation for normally distributed variables or median (range) for skewed distributions. Categorical variables were presented as patient numbers (percentage of the sample). Differences between CRBSI and non-CRBSI groups were analyzed using chi-square test or Fisher’s exact test for categorical variables, while continuous variables (e.g., age) were compared using independent samples *t*-test or Mann-Whitney U test, as appropriate. To identify significant independent risk factors for CRBSI, both univariate and multivariate analyses were conducted. Variables demonstrating a potential association with CRBSI in univariate analysis (*p* < 0.1) were selected as candidates for inclusion in the multivariate model, which was subsequently refined through stepwise forward and backward selection procedures. All statistical tests were two-tailed, with *p* < 0.05 considered statistically significant.

### 2.5 Ethical statement

This study was conducted in accordance with ethical principles for medical research involving human subjects, with strict measures to ensure patient confidentiality. Prior to commencement, the study protocol received formal approval from the institutional ethics review board (Approval No. 2025DZKY-048-01). All collected data were anonymized and used solely for the designated research objectives.

## 3 Results

### 3.1 Demographics

A total of 321 intestinal failure patients admitted between 2021 and 2024 were included in the analysis, with a median age of 55 years (range 18–79 years), comprising 190 males and 131 females. The median hospital stay was 27 days (range 7–227 days), with a median total hospitalization cost of ¥47829.49 (range ¥9827.54-242996.12).

All hospitalized patients underwent nutritional risk screening and nutritional status assessment. The NRS-2002 scores for all patients were ≥ 3, indicating nutritional risk. Among them, 79 patients (24.6%) scored 3 points, 49 (15.3%) scored 4 points, 177 (55.1%) scored 5 points, and 16 (5%) scored 6 points. According to the GLIM criteria for malnutrition diagnosis, 99 patients (30.8%) were classified as having moderate malnutrition, while 222 patients (69.2%) had severe malnutrition.

Based on the 2023 ESPEN guidelines for adult intestinal failure, the distribution of patients was 4.7% with type I, 26.8% with type II, and 68.5% with type III intestinal failure. The etiologies of intestinal failure were categorized into four groups: short bowel syndrome (217 patients, 67.6%), mechanical obstruction (57 patients, 17.8%), extensive small intestinal mucosal disease (30 patients, 9.3%), and intestinal fistula (17 patients, 5.3%). Additionally, 104 patients (32.4%) had a history of tumor, 48 (15%) had hypertension, and 36 (11.2%) had diabetes. The detailed clinical characteristics are presented in Table 1.

**TABLE 1:** Clinical characteristics of 321 IF patients.

Characteristics	Total	CRBSI	Non-CRBSI	*P*-value
n	321	66	255	< 0.001
Catheter indwelling time, median (range)	27 (4, 200)	34.5 (4, 200)	25 (4, 106)	
Gender, n (% of total cohort)				0.985
Male	190 (59.2%)	39 (12.1%)	151 (47%)	< 0.001
Female	131 (40.8%)	27 (8.4%)	104 (32.4%)	
Age (years), median (range)	55 (18, 79)	48 (18, 79)	56 (18, 74)	
BMI, median (range)	17.99 (10.24, 31.74)	18.20 (12.46, 28.90)	17.91 (10.24, 31.74)	0.472
GLIM severity, n (%)				0.915
Moderate	99 (30.8%)	20 (6.2%)	79 (24.6%)	0.448
Severe	222 (69.2%)	46 (14.3%)	176 (54.8%)	
NRS2002 score, n (%)				
4	49 (15.3%)	8 (2.5%)	41 (12.8%)	0.856
5	177 (55.1%)	42 (13.1%)	135 (42.1%)	
3	79 (24.6%)	14 (4.4%)	65 (20.2%)	
6	16 (5%)	2 (0.6%)	14 (4.4%)	
History of tumor, n (%)				
No	217 (67.6%)	44 (13.7%)	173 (53.9%)	0.115
Yes	104 (32.4%)	22 (6.9%)	82 (25.5%)	
Diabetes, n (%)				
Yes	36 (11.2%)	11 (3.4%)	25 (7.8%)	< 0.001
No	285 (88.8%)	55 (17.1%)	230 (71.7%)	
Hypertension, n (%)				
Yes	48 (15%)	21 (6.5%)	27 (8.4%)	0.185
No	273 (85%)	45 (14%)	228 (71%)	
IF cause, n (%)				
Short bowel syndrome	217 (67.6%)	43 (13.4%)	174 (54.2%)	0.118
Mechanical obstruction	57 (17.8%)	11 (3.4%)	46 (14.3%)	
Extensive mucosal disease	30 (9.3%)	5 (1.6%)	25 (7.8%)	
Intestinal fistulas	17 (5.3%)	7 (2.2%)	10 (3.1%)	
IF type, n (%)				
III	220 (68.5%)	46 (14.3%)	174 (54.2%)	0.299
II	86 (26.8%)	20 (6.2%)	66 (20.6%)	
I	15 (4.7%)	0 (0%)	15 (4.7%)	
Venous catheters, n (%)				
PICC	249 (77.6%)	48 (15%)	201 (62.6%)	0.141
PORT	14 (4.4%)	4 (1.2%)	10 (3.1%)	
PVC	42 (13.1%)	8 (2.5%)	34 (10.6%)	
CVC	16 (5%)	6 (1.9%)	10 (3.1%)	
PN frequency /wk, n (%)				
7	262 (81.6%)	58 (18.1%)	204 (63.6%)	< 0.001
< 7	59 (18.4%)	8 (2.5%)	51 (15.9%)	
Length of Stay, median (range)	27 (6, 227)	37.5 (7, 227)	24 (6, 113)	
Cost, median (range)	47,829.49 (9827.54, 242,996.12)	65,239.14 (11,241.32, 178,874.14)	48,768.90 (9827.54, 242,996.12)	< 0.001

### 3.2 Episode of CRBSI

This study retrospectively analyzed 321 hospitalized patients, encompassing 9,365 catheter-days. The median catheter indwelling time was 27 days (range 4–200 days). A total of 66 patients (20.56%) developed CRBSI, with an overall CRBSI incidence rate of 7.048 per 1,000 catheter-days.

The venous access devices utilized during the study period included: peripherally inserted central catheters (PICCs) (77.6%), implantable venous access ports (4.4%), peripheral venous catheters (PVCs) (13.1%), and central venous catheters (CVCs) (5%).

Among the 66 patients with CRBSI, the median catheter indwelling duration was 34.5 days (range 4–200 days), and the median age was 48 years (range 18–79 years). Hypertension was present in 21 patients. When stratified by intestinal failure type, CRBSI occurred in 46/220 (20.9%) patients with type III, 20/86 (23.3%) with type II, and none (0/15) with type I. By etiological classification of intestinal failure, CRBSI incidence was as follows: short bowel syndrome (43/217, 19.8%), mechanical obstruction (11/57, 19.3%), extensive small intestinal mucosal disease (5/30, 16.7%), and intestinal fistula (7/17, 41.2%). Of the 262 patients receiving daily parenteral nutrition (PN), 58 developed CRBSI. The infection rates by device type were: PICC (48/249), PORT (4/14), PVC (8/42), and CVC (6/16) (Table 1).

### 3.3 Microbiological profile of pathogens in CRBSI

A total of 75 microbial isolates were identified from blood cultures and catheter tip cultures in 66 CRBSI patients. Gram-positive bacteria were the most prevalent (41/75, 54.67%), followed by Gram-negative bacteria (28/75, 37.33%) and fungal infections (6/75, 8%). The most common pathogens were *Staphylococcus epidermidis* (*n* = 15) and *Escherichia coli* (*n* = 10). Monomicrobial infections accounted for 59 case, while polymicrobial infections were observed in 7 cases. Among polymicrobial infections, 5 cases involved 2 microbial species, and 2 cases involved 3 species. In polymicrobial infections, *Pseudomonas aeruginosa* was the most frequently identified (4/7), followed by *Enterococcus faecalis* (2/7) and *Acinetobacter baumannii complex* (2/7) (Table 2).

**TABLE 2:** Seventy five pathogens isolated in 66 patients with CRBSI.

Pathogens	Numbers (%)
Total	75
Gram positive organisms	41 (54.67)
*Staphylococcus epidermidis*	15 (20)
*Staphylococcus warneri*	5 (6.67)
*Staphylococcus hominis*	4 (5.33)
*Staphylococcus haemolyticus*	4 (5.33)
*Enterococcus faecalis*	4 (5.33)
*Enterococcus faecium*	2 (2.67)
*Staphylococcus aureus*	2 (2.67)
*Staphylococcus sciuri*	1 (1.33)
*Streptococcus agalactiae*	1 (1.33)
*Lactococcus garvieae*	1 (1.33)
*Enterococcus gallinarum*	1 (1.33)
*Proteus mirabilis*	1 (1.33)
Gram negative organisms	28 (37.33)
*Escherichia coli*	10 (13.33)
*Acinetobacter baumannii complex*	5 (6.67)
*Pseudomonas aeruginosa*	4 (5.33)
*Klebsiella pneumoniae*	3 (4)
*Flavobacterium indologenes*	2 (2.67)
*Enterobacter cloacae complex*	2 (2.67)
*Pseudomonas putida*	1 (1.33)
*Morganella morganii*	1 (1.33)
Fungi	6 (8)
*Candida parapsilosis*	4 (5.33)
*Candida albicans*	1 (1.33)
*Candida guilliermondii*	1 (1.33)

### 3.4 Risk factors for CRBSI

#### 3.4.1 Logistic regression analysis of risk factors for CRBSI

Table 3 presents the results of univariate and multivariate logistic regression analyses of risk factors for CRBSI. The univariate analysis revealed that the risk of CRBSI was significantly associated with hypertension (OR 3.941, 95% CI 2.050–7.577), PN energy proportion relative to REE > 0.6 (PN/REE > 0.6) (OR 4.238, 95% CI 2.405–7.469), daily PN volume > 1650 mL (OR 1.988, 95% CI 1.150–3.434), non-protein calorie-to-nitrogen (C/N) ratio ≥ 100 kcal/g N (OR 2.231, 95% CI 1.241–4.011), glucose-to-lipid ratio ≤ 1 (OR 1.789, 95% CI 1.037–3.087), lymphocyte count (LYC) < 1 × 10^9^/L (OR 6.325, 95% CI 3.493–11.453), and prealbumin ≤ 200 mg/L (OR 3.109, 95% CI 1.728–5.593). Subsequently, variables with p < 0.1 in the univariate analysis were incorporated into the multivariate analysis after testing for collinearity. The results demonstrated that PN/REE > 0.6 (OR 3.808, 95% CI 1.862–7.789, p < 0.001), C/N ratio ≥ 100 kcal/g N (OR 2.118, 95% CI 1.051–4.269, *p* = 0.036), hypertension (OR 4.981, 95% CI 2.255–11.005, *p* < 0.001) and LYC < 1 × 10^9^/L (OR 6.047, 95% CI 3.044–12.015, *p* < 0.001) were independent risk factors for CRBSI in patients with intestinal failure, with no evidence of multicollinearity among these variables (Table 3).

**TABLE 3:** Univariate and multivariate logistic regression analysis of risk factors for CRBSI.

Characteristics	Total (N)	Univariate analysis		Multivariate analysis		
		Odds Ratio (95% CI)	*P*-value	Odds Ratio (95% CI)	*P*-value	VIF
Age (years)	321	Reference	0.587	Reference	< 0.001	Reference
< 65	237					
≥ 65	84	1.182 (0.647–2.159)				
Sex	321					
Male	190	Reference				
Female	131	1.005 (0.580–1.743)	0.985			
NRS-2002	321					
5	177	Reference				
4	49	0.627 (0.273–1.443)	0.272			
6	16	0.459 (0.100–2.103)	0.316			
3	79	0.692 (0.353–1.357)	0.284			
History of tumor	321	Reference				
Yes	104					
No	217	0.948 (0.533–1.685)	0.856			
Diabetes	321	Reference				
No	285					
Yes	36	1.840 (0.854–3.965)	0.120			
Hypertension	321	Reference	< 0.001			
No	273					
Yes	48	3.941 (2.050–7.577)		4.981 (2.255–11.005)		1.0488
Categories of venous catheters	321	Reference		Reference	< 0.001	Reference
PORT	14					
PICC	249	0.597 (0.180–1.985)	0.400			
PVC	42	0.588 (0.146–2.366)	0.455			
CVC	16	1.500 (0.322–6.991)	0.606			
PN frequency /wk	321	Reference				
7	262					
< 7	59	0.552 (0.248–1.228)	0.145 < 0.001			
PN/REE	321					
≤ 0.6	225	Reference				
> 0.6	96	4.238 (2.405–7.469)		3.808 (1.862–7.789)		1.21
PNvolume (ml/d)	321	Reference	0.014	Reference	0.368	Reference
≤ 1650	189					
> 1650	132	1.988 (1.150–3.434)		1.387 (0.680–2.830)		1.2093
Omega3-PUFA in PN	321	Reference	0.153			
No	54					
Yes	267	0.614 (0.314–1.199)				
Glutamine in PN	321	Reference				
No	107					
C/N ratio (kcal/g N)	321	Reference	0.007	Reference	0.036	Reference
< 100	245					
≥ 100	76	2.231 (1.241–4.011)		2.118 (1.051–4.269)		1.0227
Glucose-to-lipid ratio	321	Reference	0.037	Reference	0.059	Reference
> 1	192					
≤ 1	129	1.789 (1.037–3.087)		1.905 (0.975–3.721)		1.0649
WBC (× 10∧9 /L)	321	Reference	0.551	Reference	< 0.001	Reference
≥ 4	249					
< 4	72	0.815 (0.416–1.597)				
NEUT (× 10∧9 /L)	321	Reference	0.949			
≥ 1.5	301					
< 1.5	20	0.964 (0.311–2.985)				
LYC (× 10∧9 /L)	321	Reference	< 0.001			
≥ 1	207					
< 1	114	6.325 (3.493–11.453)		6.047 (3.044–12.015)		1.0938
Anemia	321	Reference	0.085	Reference	0.375	Reference
No	80					
Yes	241	1.855 (0.918–3.750)		1.448 (0.639–3.281)		1.0383
Prealbumin (mg/L)	321	Reference	< 0.001	Reference	0.157	Reference
> 200	161					
≤ 200	160	3.109 (1.728–5.593)		1.654 (0.824–3.318)		1.079
Albumin (g/L)	321	Reference	0.124			
< 35	173					
≥ 35	148	1.532 (0.889–2.640)				

#### 3.4.2 Subgroup analyses of risk factors for CRBSI

To investigate the impact of these four independent risk factors on CRBSI across different populations, we performed covariate-adjusted subgroup analyses stratified by age, sex, GLIM-defined malnutrition severity, intestinal failure type, tumor history, hypertension, diabetes, and anemia.

**FIGURE 2:**
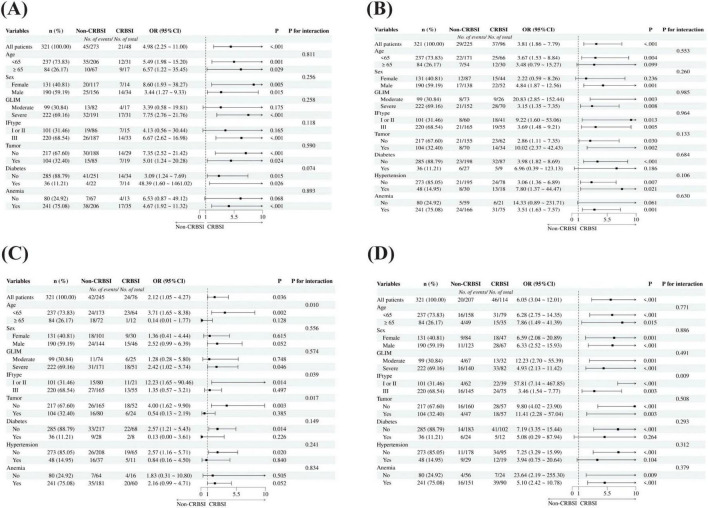
Subgroup analysis of independent risk factors for CRBSI**. (A)** Subgroup analyses of hypertension on the risk of CRBSI. **(B)** Subgroup analyses of PN/REE > 0.6 on the risk of CRBSI; **(C)** Subgroup analyses of C/N ratio ≥ 100 kcal/g N on the risk of CRBSI. **(D)** Subgroup analyses of LYC < 1 × 10^∧^9 /L on the risk of CRBSI; GLIM: Global Leadership Initiative on Malnutrition (GLIM) criteria; IF: Intestinal failure.

Patients who developed CRBSI had a significantly higher prevalence of arterial hypertension compared to those who did not (31.82% vs. 10.59%). Hypertensive patients demonstrated a significantly elevated risk of CRBSI compared to non-hypertensive counterparts. In the univariate analysis, hypertension was associated with an odds ratio of 3.941 for CRBSI. After adjusting for potential confounders such as age and diabetes in a multivariate model, the association remained significant. This association was particularly pronounced in female patients (OR 8.6, 95% CI 1.93–38.27, *p* = 0.005), those with severe malnutrition (OR 7.75, 95% CI 2.76–21.76, *p* < 0.01), type III intestinal failure (OR 6.67, 95% CI 2.62–16.98, *p* < 0.01), and non-tumor patients (OR 7.35, 95% CI 2.52–21.42, *p* < 0.01) (Figure 2A).

IF patients with higher proportion of energy supply from PN (PN/REE > 0.6) demonstrated a 3.8-fold increased risk of CRBSI compared to controls, with statistically significant association. Particularly elevated risks were observed in male patients (OR 4.84, 95% CI 1.87–12.56, *p* = 0.001), those with moderate malnutrition (OR 20.83, 95% CI 2.85–152.44, *p* = 0.003), type I/II intestinal failure (OR 9.22, 95% CI 1.60–53.06, *p* = 0.013), and patients with tumor history (OR 10.02, 95% CI 2.37–42.43, *p* = 0.002) (Figure 2B).

Patients with a C/N ratio ≥ 100 kcal/g N exhibited a 2.12-fold higher risk of CRBSI compared to controls, demonstrating statistical significance. Interaction analyses revealed significant effect modifications by age (pinteraction = 0.01), intestinal failure type (pinteraction = 0.039), and tumor history (pinteraction = 0.017). Notably, the risk was substantially elevated in patients aged < 65 years (OR 3.71, 95% CI 1.65–8.38, *p* = 0.002), those with type I/II intestinal failure (OR 12.23, 95% CI 1.65–90.46, *p* = 0.014), and non-tumor patients (OR 4.00, 95% CI 1.62–9.90, *p* = 0.003). Conversely, a potential protective effect was observed in patients aged ≥ 65 years (OR 0.14, 95% CI 0.01–1.77, *p* = 0.128) and those with tumors (OR 0.54, 95% CI 0.13–2.19, *p* = 0.385), though these associations did not reach statistical significance (Figure 2C). This finding may suggest that these two patient populations exist in a distinct metabolic state, necessitating personalized nutritional requirements; however, this observation requires further validation in future prospective studies with larger sample sizes.

Reduced lymphocyte count was significantly associated with an increased risk of CRBSI, suggesting that impaired immune function may be an important risk factor for CRBSI. The association was particularly pronounced in patients with moderate malnutrition (OR 12.23, 95% CI 2.70–55.39, *p* = 0.001), type I or II intestinal failure (OR 57.81, 95% CI 7.14–467.85, *p* < 0.001), history of tumor (OR 11.41, 95% CI 2.28–57.04, *p* = 0.003), non-hypertensive status (OR 7.25, 95% CI 3.29–15.99, *p* < 0.001), and absence of anemia (OR 23.64, 95% CI 2.19–255.30, *p* = 0.009) (Figure 2D).

#### 3.4.3 Cox regression analysis of risk factors for CRBSI

To dynamically assess the impact of risk factors on CRBSI incidence rates, we employed Cox proportional hazards models with catheter indwelling time as the time variable. As shown in Table 4, univariate Cox regression analysis revealed that patients with PN/REE > 0.6 (HR 2.755, 95% CI 1.665–4.558, *p* < 0.001), daily PN fluid volume > 1,650 mL (HR 1.862, 95% CI 1.138–3.047, *p* = 0.013), neutrophils < 1.5 × 10^9^/L (HR 3.140, 95% CI 1.843–5.348, *p* < 0.001), LYC < 1 × 10^9^/L (HR 1.934, 95% CI 1.007–3.716, *p* = 0.048), anemia (HR 1.745, 95% CI 1.009–3.020, *p* = 0.046), and albumin < 35 g/L (HR 1.886, 95% CI 1.145–3.106, *p* = 0.013) had significantly increased risk of developing CRBSI.

**TABLE 4:** Univariate and multivariate cox regression analysis of risk factors for CRBSI.

Characteristics	Total (N)	Univariate analysis		Multivariate analysis		
		Hazard ratio (95% CI)	*P*-value	Hazard ratio (95% CI)	*P*-value	VIF
Age (years)	321	Reference	0.572	Reference	0.394	Reference
< 65	237					
≥ 65	84	1.167 (0.682–1.996)				
Sex	321	Reference	0.562			
Male	190					
Female	131	0.862 (0.523–1.422)				
NRS-2002	321	Reference	0.792			
5	177					
4	49	0.902 (0.421–1.936)				
6	16	1.693 (0.399–7.187)	0.475			
3	79	1.174 (0.634–2.174)	0.610			
History of tumor	321	Reference	0.803			
Yes	104					
No	217	0.935 (0.553–1.582)				
Diabetes	321	Reference	0.552			
No	285					
Yes	36	1.221 (0.633–2.357)				
Hypertension	321	Reference	0.697			
No	273					
Yes	48	1.120 (0.633–1.982)				
Categories of Venous catheters	321	Reference	0.674			
PORT	14					
PICC	249	1.255 (0.435–3.625)				
PVC	42	2.591 (0.736–9.129)	0.138			
CVC	16	2.343 (0.644–8.531)	0.197			
PN frequency /wk	321	Reference	0.062			
7	262					
< 7	59	0.492 (0.233–1.036)		1.487 (0.597–3.701)		1.501
PN/REE	321	Reference	< 0.001	Reference	0.015	Reference
≤ 0.6	225					
> 0.6	96	2.755 (1.665–4.558)		2.055 (1.152–3.668)		1.3213
PNvolume (ml/d)	321	Reference	0.013	Reference	0.174	Reference
≤ 1650	189					
> 1650	132	1.862 (1.138–3.047)		1.466 (0.845–2.543)		1.2614
Omega3-PUFA in PN	321	Reference	0.317			
No	54					
Yes	267	0.737 (0.405–1.341)				
Glutamine in PN	321	Reference	0.575			
No	107					
Yes	214	0.861 (0.509–1.455)				
C/N ratio (kcal/g N)	321	Reference	0.081	Reference	0.125	Reference
< 100	245					
≥ 100	76	1.580 (0.946–2.640)		1.504 (0.893–2.533)		1.0415
Glucose-lipid ratio	321	Reference	0.552	Reference	0.002	Reference
> 1	249					
≤ 1	72	0.824 (0.435–1.560)				
WBC (×10∧9/L)	321	Reference	0.576			
≥ 4	301					
< 4	20	1.338 (0.482–3.715)				
NEUT (× 10∧9 /L)	321	Reference	< 0.001			
≥ 1.5	207					
< 1.5	114	3.140 (1.843–5.348)		2.573 (1.417–4.673)		1.2478
LYC (× 10∧9 /L)	321	Reference	0.048	Reference	0.075	Reference
≥ 1	80					
< 1	241	1.934 (1.007–3.716)		1.869 (0.938–3.721)		1.1111
Anemia	321	Reference	0.046	Reference	0.712	Reference
No	161					
Yes	160	1.745 (1.009–3.020)		0.890 (0.480–1.651)		1.2413
Prealbumin (mg/L)	321	Reference	0.295	Reference	0.072	Reference
> 200	173					
≤ 200	148	1.304 (0.793–2.145)				
Albumin (g/L)	321	Reference	0.013			
≥ 35	174					
< 35	147	1.886 (1.145–3.106)		1.595 (0.959–2.653)		1.0391

Variables with *p* < 0.1 were subsequently incorporated into multivariate analysis after testing for collinearity. The proportional hazards assumption was verified using Schoenfeld residual tests. The higher proportion of energy supply from PN (PN/REE > 0.6) showed a significant time-varying effect (*P* = 0.0052), indicating non-proportional hazards. To address this violation, we incorporated a time-dependent interaction term (PN/REE × time = PN/REE × ln[time]) into the model. All other covariates maintained *P* > 0.05 in the Schoenfeld tests, confirming constant hazard ratios throughout the follow-up period (Supplementary Table S1).

**FIGURE 3:**
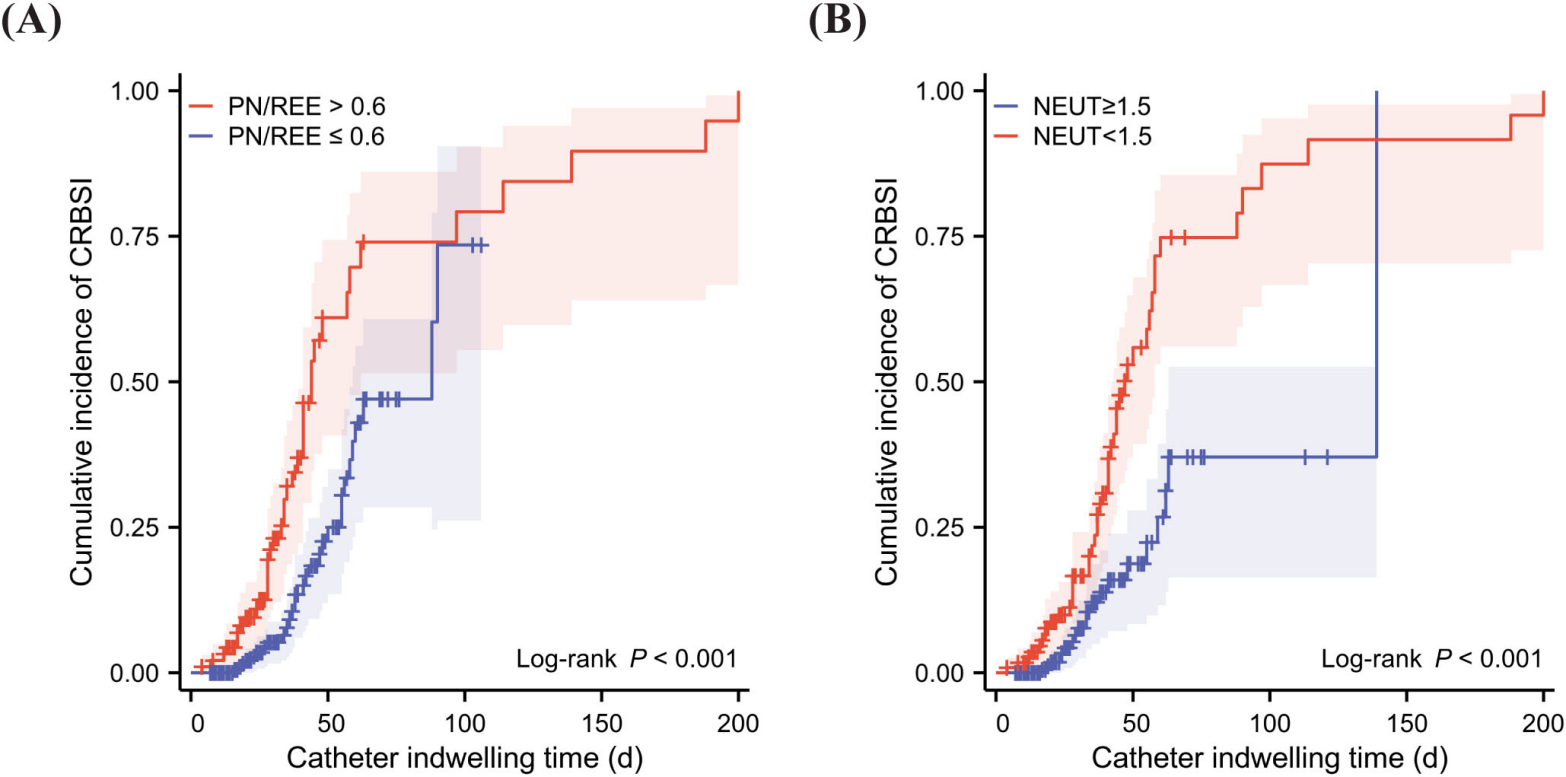
Kaplan-Meier analysis of cumulative incidence of CRBSI. **(A)** Cumulative incidence of CRBSI over time in patients with prolonged catheter indwelling, compared by PN/REE levels. **(B)** Cumulative incidence of CRBSI over time in patients with prolonged catheter indwelling, compared by the presence of neutropenia. PN, Parenteral nutrition; REE, Resting energy expenditure; PN/REE, PN energy proportion relative to REE; NEUT, Neutrophil.

The results demonstrated that PN/REE > 0.6 (HR 2.055, 95% CI 1.152–3.668) and neutrophils < 1.5 × 10^9^/L (HR 2.573, 95% CI 1.417–4.673) were independent risk factors for CRBSI in patients with intestinal failure, with no collinearity observed among the variables. Kaplan-Meier analysis demonstrated a significant increase in CRBSI incidence over time with prolonged catheter indwelling in patients with PN/REE > 0.6 and neutrophil counts < 1.5 × 10^9^/L (Log-rank test, *P* < 0.001) (Figure 3).

### 3.5 Impact of CRBSI on length of hospital stay and hospitalization costs

**FIGURE 4:**
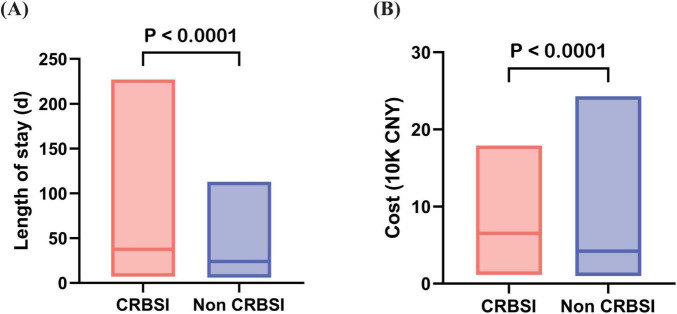
Differences in length of hospital stay and hospitalization costs between CRBSI and Non-CRBSI patients. **(A)** Comparison of length of stay between CRBSI and Non-CRBSI patients. **(B)** Comparison of hospitalization costs between CRBSI and Non-CRBSI patients.

In patients with intestinal failure, the median hospitalization duration was significantly longer in the CRBSI group compared to the Non-CRBSI group, with a difference of 13.5 days (*p* < 0.001). Furthermore, the median hospitalization costs were substantially higher in CRBSI patients versus Non-CRBSI patients, demonstrating a statistically significant difference (*p* < 0.001) (Table 1; Figure 4).

## 4 Discussion

This study provides a novel investigation into the risk factors for CRBSI among hospitalized intestinal failure patients, with particular emphasis on nutritional variables for the first time. We highlight the critical role of nutrition support in infection prevention.

### 4.1 Difference in the incidence of CRBSI compared with HPN

The CRBSI incidence rate reported in the current study was significantly higher than the previously reported rates of approximately 1–2 per 1,000 catheter-days in HPN patients (10–13). This discrepancy may be attributed to several factors, including increased nosocomial infections due to hospital-acquired pathogens, overlooked CRBSI-specific risk factors unique to hospitalized intestinal failure patients, and potential differences in susceptibility among various intestinal failure subtypes or patient subpopulations during hospitalization.

### 4.2 Distinctive characteristics of pathogen distribution during hospitalization

Among all cultured pathogens, coagulase-negative Staphylococcus epidermidis was the most predominant isolate, consistent with previous reports (4, 14, 15). *Escherichia coli*, a Gram-negative bacterium ranked as the second most prevalent pathogen, has been rarely documented in CRBSI studies of HPN patients but represents a common causative organism in hospitalized CRBSI cases (16–18). Notably, our analysis identified multiple instances of Pseudomonas aeruginosa and Acinetobacter baumannii complex in the seven polymicrobial infections. These highly resistant pathogens have been increasingly reported in CRBSI studies involving oncology, hemodialysis, hematopoietic stem cell transplantation, and ICU patients (16, 19–23). Hospitalized intestinal failure patients may demonstrate heightened susceptibility to these virulent nosocomial pathogens due to compromised immune function and impaired intestinal barrier integrity. Furthermore, the perioperative prophylactic antibiotics or gut decontamination regimens may create a favorable microenvironment for these resistant strains to proliferate, particularly in intestinal failure patients with unstable metabolic conditions. In the clinical management of CRBSI in intestinal failure patients, empirical antibiotic selection should primarily cover coagulase-negative staphylococci while remaining vigilant for Gram-negative bacteria. Additionally, heightened awareness of resistant bacterial infections in hospitalized patients is essential, and unnecessary prophylactic antibiotic use should be avoided.

### 4.3 Dual-model multivariate analysis of independent risk factors for CRBSI

Through multivariate regression analysis, we identified independent risk factors for CRBSI in IF patients. Previous studies have identified several risk factors for CRBSI in patients receiving HPN, including age, sex, catheter type, remnant small bowel length, socioeconomic status, and duration of HPN (4, 12, 13, 24, 25). This study expands beyond these conventional HPN-associated risk factors by innovatively focusing on the critical roles of nutritional modulation, immune monitoring, and individualized assessment based on intestinal failure types in CRBSI prevention during hospitalization.

PN/REE > 0.6 showed significant associations in both models, strongly validating it as a core intervenable target. In IF patients, compromised nutrient assimilation mandates PN dependence. Hospitalization exacerbates PN reliance due to suboptimal EN delivery during metabolic fluctuations. A meta-analysis by Jiang et al. demonstrated that low-calorie parenteral nutrition (PN) significantly reduced infectious complications and shortened hospital stays (26). Furthermore, two additional clinical studies established increased parenteral caloric intake as an independent risk factor for bloodstream infections in patients receiving PN (27, 28). Consistent with the above results, we established PN/REE > 0.6 as a consistent CRBSI predictor, with cumulative risk over catheter-days. This necessitates: (1) Strategic EN advancement to tolerance thresholds; (2) Rigorous PN/REE monitoring. Existing evidence corroborates EN’s role in infection reduction (29, 30) and our prior research confirms the protective effects of EN during PN-dependent periods (31, 32). Collectively, these data support a clinical strategy of gradually escalating EN volume and maintaining PN/REE ≤ 0.6 to mitigate CRBSI risk in intestinal failure patients.

### 4.4 Subtype-specific risk modulation in intestinal failure

This study is the first to demonstrate subtype-specific modulation of CRBSI risk factors in intestinal failure. Type I/II patients with a C/N ratio ≥ 100 kcal/g N and lymphocytopenia (<1 × 10^9^/L) exhibited significantly elevated CRBSI risk. The immunological vulnerability in metabolically unstable type I/II patients—exacerbated by surgical stress and metabolic dysregulation—predisposes them to immune impairment from high C/N ratios and lymphocytopenia. Elevated C/N ratios may reflect insufficient amino acid provision or excessive glucose/lipid loads, where amino acid deficiency compromises lymphocyte energy metabolism. High glucose levels induce mitochondrial damage in various critical cell types, including immune cells (33). Furthermore, studies have demonstrated that significant hyperglycemia impairs macrophage phagocytic capacity and oxidative burst activity (34). Both pathways directly contribute to immune dysfunction and CRBSI pathogenesis (33, 35).

When nutritional support provides excessive non-protein calories, particularly from an increased lipid energy load, the rapid infusion of large doses of lipid emulsions may surpass the body’s normal metabolic clearance capacity. This can amplify its interference with immune function. n-6 polyunsaturated fatty acids contribute to a pro-inflammatory immune milieu via the generation of pro-inflammatory metabolites, which may potentially compromise the host’s initial defense against pathogens. Meanwhile, medium-chain triglycerides (MCT) activate leukocytes and promote their adhesion to the vascular endothelium, potentially transporting bacteria to the catheter wall. Concurrently, lipid particles can suppress the clearance function of the reticuloendothelial system, collectively increasing the risk of CRBSI (36).

Our study identifies a significant association between arterial hypertension and CRBSI. As illustrated by the expert-derived causal diagram (DAG) (37), a patient’s baseline factors (such as comorbidities) and initial clinical status influence their risk of infection. Hypertension is closely associated with endothelial dysfunction, a chronic low-grade inflammatory state, and immune dysregulation. However, we cannot rule out residual confounding by other unmeasured or insufficiently adjusted comorbidities (e.g., chronic kidney disease or degree of immunosuppression) that may have influenced the observed association. Therefore, hypertension should not be considered a definitive independent risk factor but rather a marker requiring further investigation in studies designed to collect and adjust for these specific biochemical and comorbidity parameters. In type III patients, this endothelial dysfunction could compromise the vascular interface at the catheter site, facilitating bacterial colonization, compounded by small intestinal bacterial overgrowth (SIBO) and long-term PN-related gut barrier disruption that enhance microbial translocation (38). This “gut-vascular” interaction positions hypertension not merely as a comorbidity but as a potential indicator of compromised anti-infective defense.

Consequently, CRBSI prevention mandates metabolic monitoring and enteral nutrition optimization across all subtypes. Type I/II patients require amino acid-enriched formulations with immunonutrient to restore immune competence, whereas type III patients necessitate stringent blood pressure control and vascular integrity maintenance.

### 4.5 Limitations

The single-center, retrospective design represents a key limitation of this study, potentially restricting the generalizability of our findings to broader populations. While conducted at China’s largest center for this rare condition in adults, this design inherently limits patient diversity and external validity. Another important limitation is that adherence to standardized catheter care and aseptic maintenance protocols could not be guaranteed or objectively verified. Differences in clinical practice are potential unmeasured confounders that may have independently influenced the observed CRBSI rates. The lack of continuous glucose monitoring data precludes an analysis of glycemic control as a risk factor. This is a salient gap in our study that merits focused investigation in future prospective cohorts. Also, the lack of dynamic monitoring of key metabolic biomarkers hindered in-depth exploration of the molecular pathways linking high calorie-to-nitrogen ratios to immune impairment.

Based on the limitations identified, future prospective studies should adopt a multicenter design to enhance the generalizability of findings. It is crucial to incorporate objective monitoring of catheter care adherence, continuous glucose levels, and dynamic metabolic biomarkers. These data will allow for rigorous adjustment of confounders and enable in-depth exploration of the biological mechanisms, such as how substrate metabolism influences immunity. Ultimately, nutritional intervention trials are needed to validate the efficacy of tailored nutritional strategies in reducing infection risk.

## 5 Conclusion

Hospitalized intestinal failure patients face unique risk factors for CRBSI, necessitating vigilance against multidrug-resistant nosocomial pathogens alongside regular monitoring of energy metabolism and adjustments to parenteral nutrition formulations. We recommend a combined nutritional strategy for intestinal failure patients: strategically advance enteral nutrition to tolerance while rigorously monitoring and maintaining the PN/REE ratio at or below 0.6. For type I/II IF patients, we recommend a low calorie-to-nitrogen ratio PN formula with immunonutrients. For type III, blood pressure management remains a vital focus. This study highlights the critical role of tailored nutritional support in mitigating CRBSI risk among hospitalized IF patients, with subtype-specific strategies offering promising directions for future clinical practice.

## Data Availability

The datasets presented in this article are not readily available due to privacy and ethical restrictions. Requests to access the datasets should be directed to the corresponding author.

## Footnotes

1
www.zstats.net
