# Supplementary material for: Nutrition-modulated, subtype-specific risk factors for catheter-related bloodstream infections in hospitalized patients with intestinal failure
Source: Front Nutr. 2025 Nov 20;12:1705357. doi: 10.3389/fnut.2025.1705357 (PMC12675214; doi:10.3389/fnut.2025.1705357)
Supplement: Supplementary file 1 [file Table_1.docx]

**Table S1.** Schoenfeld residuals test for variables in multivariate analysis

| Variables | Chi-square | P-value |
| --- | --- | --- |
| PN frequency per week | 0.26162 | 0.6090 |
| PN/REE | 7.797 | 0.0052 |
| PN volume per day | 1.2705 | 0.2597 |
| Energy nitrogen ratio | 1.0396 | 0.3079 |
| NEUT(×10^9 /L) | 0.24229 | 0.6226 |
| LYC (×10^9 /L) | 2.3728 | 0.1235 |
| Anemia | 0.21567 | 0.6424 |
| Albumin (g/L) | 0.13751 | 0.7108 |
| GLOBAL | 11.686 | 0.1658 |
